# Postoperative Inflammatory Markers in Older Adults: A Longitudinal Study of Delirium After Spine Surgery

**DOI:** 10.1093/geroni/igaf122.2320

**Published:** 2025-12-31

**Authors:** Jeongeun Choi, Hyangkyu Lee

**Affiliations:** Yonsei University, Seoul, Republic of Korea; Yonsei University, Seoul, Republic of Korea

## Abstract

Systemic inflammation is linked to postoperative delirium (POD) and adverse postoperative outcomes in older adults undergoing spine surgery. Previous studies have primarily focused on cytokine changes, limiting clinical applicability. This study aimed to examine changes in blood-based clinical inflammatory biomarker levels over time in older adults with and without POD. This study included 536 participants aged 70 and older who underwent elective spine surgery between November 2019 and May 2023. Serum inflammatory markers, including the neutrophil-to-lymphocyte ratio (NLR), platelet-to-lymphocyte ratio (PLR), systemic immune-inflammation index (SII), and C-reactive protein (CRP), were measured at three time points: pre-operation (Time 1), postoperative day 1 (Time 2), and discharge (Time 3). The trajectories of these markers were analyzed using linear mixed models, adjusting for age, sex, body mass index, Charlson Comorbidity Index (CCI), American Society of Anesthesiologists (ASA) score, operative level, and baseline inflammatory marker levels. 95 participants (17.7%) out of 536 developed POD. Compared to the non-delirium group, participants with delirium exhibited significantly higher inflammatory marker levels with different patterns. In particular, NLR and SII levels were significantly elevated at Time 2 (both p < 0.001), while PLR levels were elevated at both Time 2 (p < 0.001) and Time 3 (p = 0.021), and CRP levels gradually increased over time and remained high at Time 3 (p = 0.006). These findings suggest that elevated inflammatory marker levels in older adults experiencing POD persist until discharge, highlighting the need for timely monitoring to reduce delirium risk and improve postoperative recovery.

